# Regression trees for predicting mortality in patients with cardiovascular disease: What improvement is achieved by using ensemble-based methods?

**DOI:** 10.1002/bimj.201100251

**Published:** 2012-07-06

**Authors:** Peter C Austin, Douglas S Lee, Ewout W Steyerberg, Jack V Tu

**Affiliations:** 1 Institute for Clinical Evaluative SciencesToronto, Ontario, Canada; 2 Institute of Health Policy, Management and Evaluation, University of TorontoToronto, Canada; 3 Dalla Lana School of Public Health, University of TorontoToronto, Canada; 4 Division of Cardiology, Sunnybrook Schulich Heart Centre and Faculty of Medicine, University of TorontoToronto, Canada; 5 Peter Munk Cardiac Centre and the Department of Medicine, University Health Network and Faculty of Medicine, University of TorontoToronto, Canada; 6 Department of Public Health, Erasmus Medical CentreThe Netherlands

**Keywords:** Acute myocardial infarction, Bagging, Boosting, Data mining, Heart failure

## Abstract

In biomedical research, the logistic regression model is the most commonly used method for predicting the probability of a binary outcome. While many clinical researchers have expressed an enthusiasm for regression trees, this method may have limited accuracy for predicting health outcomes. We aimed to evaluate the improvement that is achieved by using ensemble-based methods, including bootstrap aggregation (bagging) of regression trees, random forests, and boosted regression trees. We analyzed 30-day mortality in two large cohorts of patients hospitalized with either acute myocardial infarction (*N* = 16,230) or congestive heart failure (*N* = 15,848) in two distinct eras (1999–2001 and 2004–2005). We found that both the in-sample and out-of-sample prediction of ensemble methods offered substantial improvement in predicting cardiovascular mortality compared to conventional regression trees. However, conventional logistic regression models that incorporated restricted cubic smoothing splines had even better performance. We conclude that ensemble methods from the data mining and machine learning literature increase the predictive performance of regression trees, but may not lead to clear advantages over conventional logistic regression models for predicting short-term mortality in population-based samples of subjects with cardiovascular disease.

## 1 Introduction

Predicting the probability of the occurrence of a binary outcome or event is of key importance in many areas of clinical and health services research. Accurate prediction of the probability of patient outcomes, such as mortality, allows for effective risk stratification of subjects and for the comparison of health care outcomes across different providers. Logistic regression is the most commonly used method for prediction in the biomedical literature.

Many clinical investigators are interested in the use of regression trees to predict the probability of the occurrence of an event. Despite studies highlighting the inferior predictive accuracy of regression trees compared to that of logistic regression (Ennis et al., 1998; Austin 2007), some authors continue to express enthusiasm for the use of regression trees (Young and Andrews, 2008). In the data mining and machine learning literature, extensions of classical regression trees have been developed. Many of these methods involve aggregating predictions over an ensemble of regression trees. These methods include bootstrap aggregated (bagged) regression trees, random forests, and boosted regression trees. However, there is a paucity of research into the comparative performance of these methods for predicting clinical outcomes.

The objective of the current study was to compare the relative performance of regression trees, ensemble-based methods, and logistic regression for predicting short-term mortality in population-based samples of patients hospitalized with cardiovascular disease.

## 2 Methods

### 2.1 Data sources

The Enhanced Feedback for Effective Cardiac Treatment (EFFECT) Study is an initiative to improve the quality of care for patients with cardiovascular disease in Ontario (Tu et al., 2004, 2009). During the first phase (referred to as the EFFECT Baseline sample), detailed clinical data were collected on patients hospitalized with acute myocardial infarction (AMI) and congestive heart failure (CHF) between April 1, 1999 and March 31, 2001 at 86 hospital corporations in Ontario, Canada, by retrospective chart review. During the second phase (referred to as the EFFECT Follow-up sample), data were abstracted on patients hospitalized with these conditions between April 1, 2004 and March 31, 2005 at 81 Ontario hospital corporations. Data on patient demographics, vital signs and physical examination at presentation, medical history, and results of laboratory tests were collected for these samples.

In the EFFECT study, data were available on 11,506 and 7889 patients hospitalized with a diagnosis of AMI during the first and second phases of the study, respectively (9945 and 8339 for CHF, respectively). After excluding subjects with missing data on key variables, 9298 and 6932 subjects were available from the first and second phases, respectively (8240 and 7608 for CHF, respectively), for inclusion in the current study.

In the current study, the outcome was a binary variable denoting whether the patient died within 30 days of hospital admission. Candidate predictor variables were those variables described in the tables in the appendices.

### 2.2 Statistical methods for predicting cardiovascular outcomes

We used conventional regression trees, bagged regression trees, random forests, and boosted regression trees to predict the probability of 30-day mortality for patients hospitalized with cardiovascular disease. Readers are referred elsewhere for details on these tree-based methods (Clark and Pregibon, 1993; Freund and Schapire, 1996; Breiman et al., 1998; Friedman et al., 2000; Breiman, 2001; Hastie et al., 2001; McCaffrey et al., 2004; Buhlmann and Hathorn, 2007).

For bagged regression trees, a regression tree was grown in each of 100 bootstrap samples. For random forests, 500 regression trees were grown. When fitting random forests of regression trees, we let the size of the set of randomly selected predictor variables used for determining each binary split to be 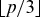, where *p* denotes the total number of predictor variables and 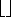 denotes the floor function (this is the default in the R implementation of random forests). For boosted regression trees, we considered four different base regression models: regression trees of depth one through four (which have also been referred to as regression trees with interaction depths one through four). For boosted regression trees, we considered sequences of 10,000 regression trees.

For all methods, we used implementations available in R statistical software (R version 2.11.1, R Foundation for Statistical Computing, Vienna, Austria). We grew conventional regression trees using the 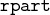 function from the *rpart* package (version 3.1-46). The optimal size of each regression tree was determined using cross-validation using the 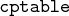 function. Regression trees were then pruned using the 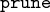 function. For bagging, random forests, and boosted regression trees, we used the 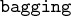 function from the *ipred* package (version 0.8-8), the 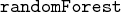 function from the *randomForest* package (version 4.5-36), and the 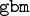 function from the *gbm* package (version 1.6-3.1), respectively.

We used two different logistic regression models to predict the probability of 30-day mortality, both of which consisted of only main effects. In the first logistic regression model, all continuous covariates were assumed to have a linear relationship with the log-odds of death. The second logistic regression model used restricted cubic smoothing splines with four knots and three degrees of freedom to model the relationship between continuous covariates and the log-odds of death (Harrell, 2001). For both logistic regression models, all candidate predictors were included in the regression models, and no variable reduction was used. We used the 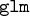 function to estimate the first logistic regression model, while we used the 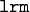 and 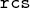 functions from the *Design* library (version 2.3-0) to estimate the logistic regression model that incorporated restricted cubic smoothing splines.

For comparative purposes, we compared the predictive performance of the above methods with previously developed disease-specific mortality prediction models. The GRACE (Global Registry of Acute Coronary Events) score was derived and validated for predicting mortality in patients hospitalized with acute coronary syndromes (Granger et al., 2003). The score comprises the following variables: Killip Class, systolic blood pressure, heart rate, age, and creatinine level. In the AMI sample, 30-day mortality was regressed on the GRACE score using a univariable logistic regression model (instead of entering the components of the score separately). We used the GRACE score as it has been shown in a recent systematic review to predict mortality in patients with acute coronary syndromes more accurately than other scores (D'Ascenzo et al., 2012). The EFFECT-HF mortality prediction model is a logistic regression model that has been derived and validated for predicting 30-day and one-year mortality in patients hospitalized with CHF (Lee et al., 2003). The model for predicting 30-day mortality uses the following variables: age, systolic blood pressure, respiratory rate, sodium, urea, history of stroke or transient ischemic attack, dementia, chronic obstructive pulmonary disease, cirrhosis of the liver, and cancer. In the CHF sample, 30-day mortality was regressed on the individual variables in the EFFECT-HF model.

### 2.3 Determining the predictive ability of different regression methods

We examined both the in-sample and out-of-sample predictive accuracy of each method. First, each model was estimated in the EFFECT Baseline sample. Using the fitted model, predictions for each subject were used to calculate the area under the receiver operating characteristic (ROC) curve (abbreviated as the AUC and which is equivalent to the *c*-statistic (Harrell, 2001; Steyerberg, 2009)), the Scaled Brier's Score, and the generalized *R^2^* index (Harrell, 2001; Steyerberg, 2009; Steyerberg et al., 2010) (the Scaled Brier Score is Brier's Score scaled by its maximum possible score). We used bootstrap methods, with 100 bootstrap samples, to calculate an optimism-corrected estimate of each measure of predictive accuracy (Efron and Tibshirani, 1993; Steyerberg, 2009). Second, we assessed model performance using the EFFECT Baseline sample as the derivation sample and the EFFECT Follow-up sample as the validation.

### 2.4 Assessing calibration

We assessed the calibration of predictions obtained in the EFFECT Follow-up sample (the validation sample) using models developed in the EFFECT Baseline sample (the derivation sample) in three different ways. First, the mean predicted probability of death in the validation sample was compared with the observed probability of death in the validation sample to indicate calibration-in-the-large (Steyerberg, 2009). Second, we determined the calibration slope (deviation of the calibration slope from unity denotes miscalibration) (Steyerberg, 2009). The calibration slope assesses deviation between observed and expected probabilities of mortality across the range of predicted risk. It may be used to indicate whether there is a need to shrink predicted probabilities. Third, using the subjects from the validation sample, we used a lowess scatterplot smoother to graphically describe the relationship between observed and predicted mortality (Harrell, 2001; Steyerberg, 2009). Deviation of this calibration plot from a diagonal line with unit slope indicates miscalibration.

### 2.5 The relationship between continuous predictor variables and the log-odds of mortality

A potential limitation to the use of regression trees is their dichotomization of continuous predictor variables. We examined the relationship between five continuous predictor variables (age, systolic blood pressure, heart rate, glucose, and creatinine) and the log-odds of 30-day mortality in the EFFECT-AMI Baseline sample. For age, we created a synthetic dataset in which age was allowed to take on the percentiles of the distribution of age in the EFFECT Baseline sample, with the value of all the other covariates in this synthetic dataset being set to the sample median in the EFFECT Baseline sample. We used each of the prediction models that were developed in the EFFECT Baseline sample to estimate the log-odds of 30-day mortality for each subject in this synthetic dataset. We repeated this process for the other four continuous variables.

## 3 Results

### 3.1 AMI sample

The percentage of patients who died within 30 days of admission did not differ between the EFFECT Baseline sample (10.9%) and the EFFECT Follow-up sample (10.5%) (*p* = 0.427, Appendices A and B).

#### 3.1.1 Comparison of predictive ability of different methods

Regression trees resulted in predicted probabilities of 30-day mortality with the lowest accuracy (Table 1). In the EFFECT Baseline sample, the use of boosted regression trees of depth four resulted in predictions with the greatest accuracy when using the AUC and the Scaled Brier's Score to assess model performance. However, a logistic regression model that incorporated restricted cubic smoothing splines resulted in the greatest out-of-sample predictive accuracy when using the EFFECT Follow-up sample as the validation sample.

**Table 1:** Measures of predictive accuracy in the AMI samples

Model	Apparent performance (EFFECT Baseline)	Optimism (bootstrap estimate)	Optimism- corrected performance (EFFECT Baseline)	EFFECT Follow- up
AUC				
Regression tree	0.768	0.013	0.755	0.767
Bagged trees	0.807	−0.005	0.812	0.820
Random forests	0.823	−0.003	0.826	0.843
Boosted trees—depth one	0.850	0.009	0.841	0.841
Boosted trees—depth two	0.864	0.013	0.851	0.848
Boosted trees—depth three	0.870	0.016	0.854	0.851
Boosted trees—depth four	0.875	0.019	0.855	0.852
Logistic regression	0.853	0.005	0.848	0.852
Logistic regression—Splines	0.862	0.009	0.854	0.858
Logistic regression—GRACE score	0.828	0.001	0.827	0.826
*R^2^*				
Regression tree	0.215	0.028	0.186	0.203
Bagged trees	0.254	−0.001	0.254	0.257
Random forests	0.288	−0.003	0.291	0.304
Boosted trees—depth one	0.324	0.021	0.304	0.295
Boosted trees—depth two	0.349	0.034	0.316	0.301
Boosted trees—depth three	0.367	0.046	0.320	0.305
Boosted trees—depth four	0.383	0.059	0.324	0.307
Logistic regression	0.332	0.012	0.320	0.315
Logistic regression—Splines	0.354	0.021	0.332	0.330
Logistic regression—GRACE score	0.280	0.001	0.279	0.259
Scaled Brier's score				
Regression tree	0.147	0.028	0.119	0.119
Bagged trees	0.168	0.001	0.167	0.119
Random forests	0.103	−0.039	0.142	0.134
Boosted trees—depth one	0.212	0.014	0.198	0.186
Boosted trees—depth two	0.246	0.027	0.219	0.197
Boosted trees—depth three	0.264	0.039	0.225	0.198
Boosted trees—depth four	0.280	0.051	0.229	0.197
Logistic regression	0.228	0.012	0.216	0.198
Logistic regression—Splines	0.246	0.021	0.225	0.211
Logistic regression—GRACE score	0.183	0.002	0.182	0.149

**Figure 1:**
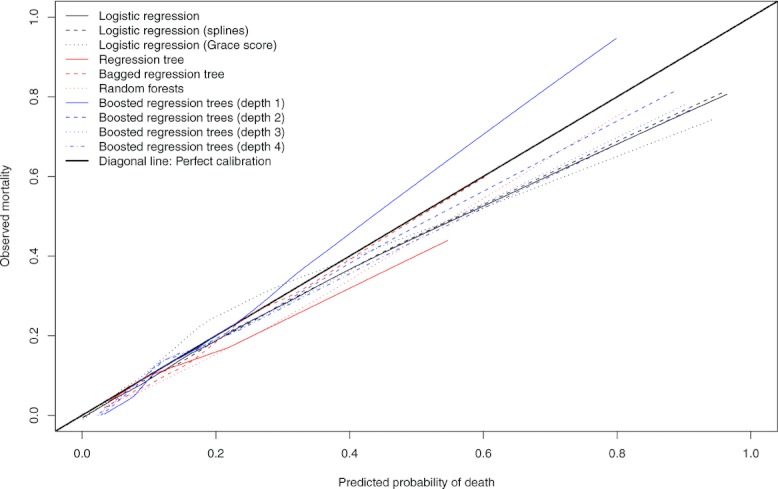
Calibration plot in EFFECT2 AMIcohort.

The three logistic regression models, random forests, and boosted regression trees of depth four resulted in calibration slopes closest to one (Table 2). The two logistic regression models had very similar calibration to one another (Fig. 1). The calibration of the GRACE risk score model deviated from that of the other two logistic regression models in the upper range of predicted risk. The regression tree resulted in predictions that displayed the greatest degree of miscalibration. Apart from boosted regression trees of depth one, the remaining prediction methods resulted in some overestimation of the risk of death among subjects with a higher predicted probability of death. Of the four boosted regression trees, the use of trees of depth two resulted in predictions with the best calibration. No method had uniformly superior calibration compared to the other approaches. Logistic regression (with or without splines) demonstrated good concordance between observed and predicted probabilities among subjects with a lower predicted probability of death. However, bagged regression trees and random forests resulted in predictions with a good concordance between observed and predicted probabilities among subjects with a higher predicted probability of death. To a certain extent, the use of boosted regression trees of depth two resulted in reasonable performance across the range of predicted values.

**Table 2:** Measures of model calibration in the EFFECT Follow-up samples

Model	AMI Cohort		CHF Cohort	
		
	Calibration intercept	Calibration slope	Calibration intercept	Calibration slope
Logistic regression	−0.171	1.000	−0.091	1.032
Logistic regression—GRACE score/	0.158	1.045	−0.118	1.029
EFFECT-HF model				
Logistic regression—splines	−0.181	0.985	−0.189	0.985
Regression tree	−0.395	0.896	−0.343	0.890
Bagged regression tree	0.073	1.174	0.273	1.215
Random forest	−0.287	1.022	−0.360	0.950
Boosted trees—depth one	0.505	1.410	0.612	1.407
Boosted trees—depth two	0.029	1.144	0.270	1.230
Boosted trees—depth three	−0.098	1.074	0.117	1.149
Boosted trees—depth four	−0.155	1.040	0.042	1.108

#### 3.1.2 Continuous predictor variables and the log-odds of mortality

**Figure 2:**
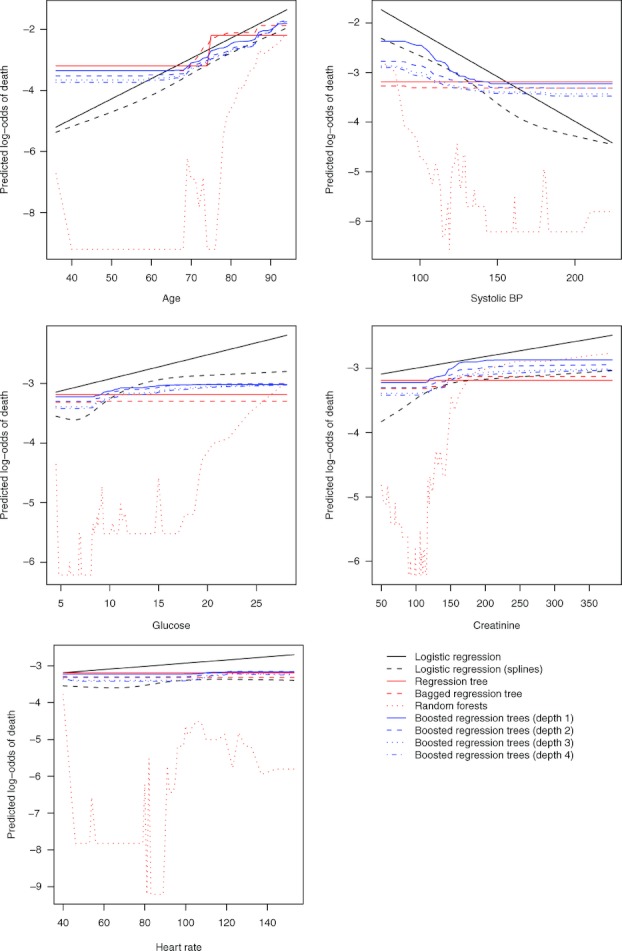
Relationship between key continuous variables and log-odds of death.

The relationship between age and the log-odds of death was approximately linear according to the restricted cubic smoothing splines (Fig. 2). The regression tree modeled a single step function to relate age to the log-odds of the outcome. The ensemble-based methods described a flat relationship between age and the log-odds of the outcome until approximately age 70 years, at which point, the log-odds of death increased with increasing age. For each of the four other covariates, the regression tree modeled a flat or null relationship between the covariate and the log-odds of death. Either the covariate was not used in the regression tree, or it was used in only a branch of the tree that was different from that branch of the tree that described the subject whose covariates were set to the sample median. Furthermore, for some of the covariates (e.g., heart rate and creatinine), the logistic regression model that incorporated restricted cubic splines described a relationship that was approximately flat at the lower range of the distribution of the covariate and/or was approximately flat at the higher range of the distribution of the covariate. Several of the ensemble-based methods approximated these plateau-like relationships.

#### 3.1.3 The distributions of predicted risks

**Figure 3:**
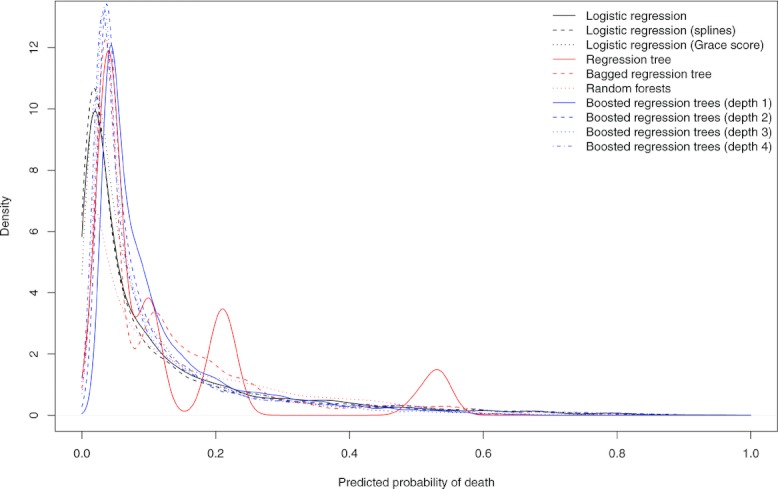
Distribution of predicted probabilities of death in AMI sample.

We report nonparametric estimates of the distribution of the predicted probability of 30-day death for each subject in the validation sample using each of the different prediction methods (Fig. 3). Since the fitted regression tree had eight terminal nodes, there were only eight different predicted probabilities of 30-day death. Apart from regression trees and bagged regression trees, the other predictive models provided unimodal distributions of predicted risk. Furthermore, the distributions were, as would be expected clinically, positively skewed. Logistic regression resulted in predicted probabilities of 30-day death that ranged from 0.001 to 0.964 (0.001–0.961 when smoothing splines were incorporated into the model). When a conventional regression tree was used, the range of predicted probabilities was 0.040–0.546. With boosted regression trees of depth four, the range was 0.023–0.907.

### 3.2 CHF sample

The percentage of subjects who died within 30 days of admission did not differ between the EFFECT Baseline sample (10.8%) and the EFFECT Follow-up sample (9.9%) (*p* = 0.083, Appendices C and D).

#### 3.2.1 Comparison of predictive ability of different regression methods

For all three measures of predictive accuracy, regression trees resulted in predicted probabilities of 30-day mortality with both the lowest in-sample and out-of-sample accuracy (Table 3). In the EFFECT Baseline sample, the use of boosted regression trees of depth four resulted in predictions with the greatest accuracy when assessing performance using the AUC and the Scaled Brier's Score. A logistic regression model that incorporated restricted cubic smoothing splines resulted in the greatest out-of-sample predictive accuracy when using the EFFECT Follow-up sample as the validation sample.

**Table 3:** Measures of accuracy in CHF samples

Model	Apparent performance (EFFECT Baseline)	Optimism (bootstrap estimate)	Optimism- corrected performance (EFFECT Baseline)	EFFECT Follow- up
AUC				
Regression tree	0.674	0.012	0.662	0.661
Bagged trees	0.713	−0.011	0.724	0.725
Random forests	0.752	−0.003	0.755	0.764
Boosted trees—depth one	0.769	0.012	0.757	0.760
Boosted trees—depth two	0.788	0.021	0.767	0.770
Boosted trees—depth three	0.801	0.029	0.772	0.774
Boosted trees—depth four	0.811	0.036	0.776	0.777
Logistic regression	0.773	0.008	0.765	0.781
Logistic regression—Splines	0.786	0.013	0.773	0.786
Logistic regression—EFFECT HF	0.762	0.003	0.759	0.775
*R^2^*				
Regression tree	0.096	0.018	0.079	0.077
Bagged trees	0.119	−0.003	0.122	0.117
Random forests	0.164	−0.007	0.171	0.170
Boosted trees—depth one	0.187	0.019	0.168	0.163
Boosted trees—depth two	0.220	0.040	0.180	0.175
Boosted trees—depth three	0.244	0.060	0.184	0.178
Boosted trees—depth four	0.266	0.079	0.187	0.180
Logistic regression	0.194	0.012	0.182	0.194
Logistic regression—Splines	0.216	0.022	0.194	0.203
Logistic regression—EFFECT HF	0.174	0.004	0.170	0.179
Scaled Brier's score				
Regression tree	0.058	0.016	0.043	0.039
Bagged trees	0.071	−0.001	0.071	0.039
Random forests	0.097	−0.021	0.118	0.087
Boosted trees—depth one	0.106	0.010	0.096	0.091
Boosted trees—depth two	0.139	0.026	0.113	0.104
Boosted trees—depth three	0.161	0.040	0.121	0.106
Boosted trees—depth four	0.179	0.054	0.126	0.107
Logistic regression	0.125	0.010	0.115	0.113
Logistic regression—Splines	0.142	0.018	0.124	0.119
Logistic regression—EFFECT HF	0.106	0.004	0.103	0.098

Boosted regression trees of depth four resulted in the mean predicted log-odds of death being the closest to the observed log-odds of death in the validation sample (Table 2). The three logistic regression models resulted in calibration slopes closest to one.

**Figure 4:**
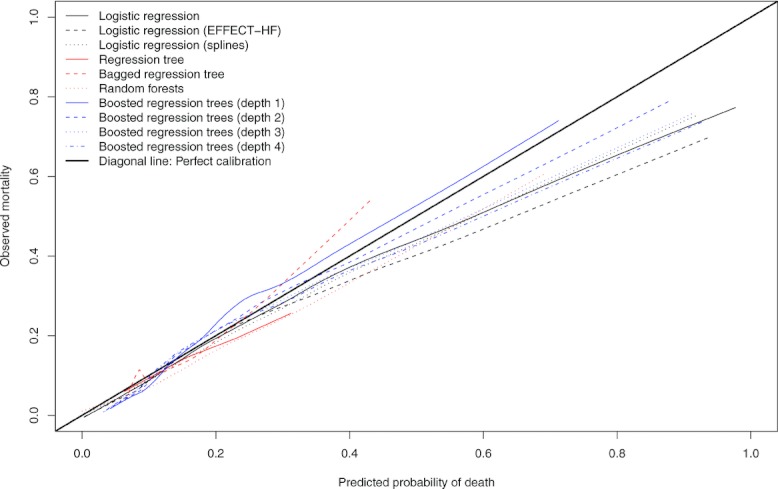
Calibration plot in EFFECT2 CHF cohort.

As in the AMI sample, no method had uniformly superior calibration to the other methods (Fig. 4). Logistic regression (with or without splines) and random forests resulted in predictions with a good concordance between observed and predicted probabilities among subjects with a lower predicted probability of death.

## 4 Discussion

We examined the ability of ensemble-based methods to predict the probability of 30-day mortality in patients who were hospitalized with either an AMI or CHF. Our primary finding was that logistic regression models that incorporated restricted cubic smoothing splines had the greatest out-of-sample predictive accuracy, in both the AMI and CHF populations. Our derivation and validation samples consisted of population-based samples of unselected patients with either AMI or CHF from temporally distinct periods (1999–2001 vs. 2004–2005, respectively). Patients in the validation sample tended to be older and modestly sicker than patients in the derivation sample. For these reasons, the estimates of out-of-sample performance are likely to be generalizable to other current settings.

Several secondary findings should be highlighted from the current study. First, ensemble-based methods offer substantially greater predictive accuracy compared to conventional regression trees for predicting short-term mortality in patients hospitalized with cardiovascular disease. Second, for predicting short-term cardiovascular mortality, ensemble-based methods did not offer a clear advantage over conventional logistic regression. Third, logistic regression resulted in the greatest range of predicted probabilities of 30-day death in the validation sample. Logistic regression thus permitted for the greatest degree in separation of patients according to predicted probability.

In the current study, we have focused on predicting outcomes rather than on describing the nature of the relationship between specific covariates and the outcome. While the latter is of interest in clinical medicine and epidemiology, prediction is also of great importance. First, it allows clinicians to make treatment decisions informed by global patient prognosis instead of multiple potential clinical factors that may have variable impacts on mortality risk. It has been previously demonstrated that without the guidance of global risk scores, the prescription of drug therapies demonstrates a risk-treatment mismatch, such that higher-risk patients are less likely to receive potentially life-saving treatment (Lee et al., 2005). Ideally, prognostic data should guide treatment decisions because: (a) some treatments should be restricted to patients with a poor prognosis, considering side effects of treatment and financial costs (e.g., coronary artery bypass graft surgery); (b) conversely, patients with a poor prognosis may not be candidates for other therapies (e.g., implantable cardiac defibrillators); (c) the timing of different treatment options versus end-of-life care is dependent on prognosis; and (d) admission to hospital is ideally reserved for patients who have worse prognosis (Lee et al., 2010).

When assessing prognosis, multivariate risk scores such as the GRACE score or the EFFECT-HF model have several potential advantages for clinicians, administrators, and researchers. They allow physicians to synthesize information from multiple clinical characteristics (e.g., demographic, vital signs, laboratory measurements, presenting signs and symptoms) to make global predictions about prognosis, rather than being overly influenced by subjective interpretation of specific patient characteristics in isolation. Thus, the models developed in this study synthesize information to improve the accuracy of the prediction of patient prognosis. Furthermore, risk models are essential for risk adjustment when comparing quality of care and outcomes among different health care plans and providers (i.e., hospital report cards). Finally, the design and analysis of randomized controlled trials may benefit from stratification by prognosis (Steyerberg, 2009). While the extent of clinical use is not definitively known, the GRACE score appears to be commonly used as research tool for formally determining patient risk in the context of research studies, rather than as a tool for clinical decision making. Widespread adoption of these risk scores and of models similar to those developed in the current study by clinicians could improve the ability of physicians to make estimates of patients’ prognosis, rather than relying on a subjective interpretation of specific clinical characteristics.

Some limitations of our study need to be acknowledged. We applied only a selection of modern modeling methods. Regression models did not include shrinkage or penalized estimation methods. We did not consider neural networks, support vector machine techniques, or the recently proposed “superlearner”, which may be relevant alternative approaches in some circumstances (van der Laan and Rose, 2011).

We conclude that bagged regression trees, random forests, and boosted regression trees may result in superior prediction of 30-day mortality in AMI and CHF patients compared to conventional regression trees. However, ensemble-based prediction methods may not offer improvements over logistic regression models that incorporated flexible functions to model nonlinear relationships between continuous covariates and the log-odds of the outcome.

## Appendix

**Appendix A:** Comparison of baseline characteristics between AMI patients who died within 30 days of admission and those who survived for 30 days subsequent to admission in the EFFECT Baseline and Follow-up samples

Variable	EFFECT Baseline sample Death within 30 days		*p*-value	EFFECT Follow-up sample Death within 30 –days		*p*-value
				
	No (*N* = 8288)	Yes (*N* = 1010)		No (*N* = 6206)	Yes (*N* = 726)	
Age	68.0 (56.0–77.0)	80.0 (73.0–86.0)	<.001	68.0 (56.0–78.0)	82.0 (74.0–87.0)	<.001
Female sex	2837 (34.2%)	496 (49.1%)	<.001	2209 (35.6%)	354 (48.8%)	<.001
Cardiogenic shock	50 (0.6%)	92 (9.1%)	<.001	6 (0.1%)	14 (1.9%)	<.001
Acute congestive heart failure/pulmonary edema	390 (4.7%)	136 (13.5%)	<.001	369 (5.9%)	110 (15.2%)	<.001
Systolic blood pressure	148.0 (129.0–170.0)	128.5 (106.0–150.0)	<.001	144.0 (125.0–165.0)	123.0 (104.0–146.0)	<.001
Diastolic blood pressure	83.0 (71.0–96.0)	72.5 (60.0–88.0)	<.001	80.0 (70.0–93.0)	70.0 (58.0–84.0)	<.001
Heart rate	80.0 (67.0–96.0)	90.0 (72.0–111.0)	<.001	81.0 (68.0–97.0)	90.0 (74.0–109.0)	<.001
Respiratory rate	20.0 (18.0–22.0)	22.0 (20.0–28.0)	<.001	20.0 (18.0–21.0)	20.0 (18.0–26.0)	<.001
Diabetes	2094 (25.3%)	339 (33.6%)	<.001	1683 (27.1%)	249 (34.3%)	<.001
Hypertension	3793 (45.8%)	493 (48.8%)	0.067	3599 (58.0%)	450 (62.0%)	0.039
Current smoker	2815 (34.0%)	195 (19.3%)	<.001	1777 (28.6%)	100 (13.8%)	<.001
Dyslipidemia	2676 (32.3%)	183 (18.1%)	<.001	2821 (45.5%)	266 (36.6%)	<.001
Family history of CAD	2693 (32.5%)	135 (13.4%)	<.001	2096 (33.8%)	91 (12.5%)	<.001
Cerebrovascular disease/TIA	761 (9.2%)	188 (18.6%)	<.001	673 (10.8%)	183 (25.2%)	<.001
Angina	2715 (32.8%)	358 (35.4%)	0.086	1823 (29.4%)	276 (38.0%)	<.001
Cancer	234 (2.8%)	51 (5.0%)	<.001	94 (1.5%)	22 (3.0%)	0.003
Dementia	239 (2.9%)	129 (12.8%)	<.001	265 (4.3%)	126 (17.4%)	<.001
Peptic ulcer disease	459 (5.5%)	56 (5.5%)	0.993	285 (4.6%)	62 (8.5%)	<.001
Previous AMI	1863 (22.5%)	280 (27.7%)	<.001	1430 (23.0%)	242 (33.3%)	<.001
Asthma	452 (5.5%)	62 (6.1%)	0.368	384 (6.2%)	43 (5.9%)	0.779
Depression	571 (6.9%)	105 (10.4%)	<.001	593 (9.6%)	102 (14.0%)	<.001
Peripheral vascular disease	593 (7.2%)	119 (11.8%)	<.001	488 (7.9%)	107 (14.7%)	<.001
Previous revascularization	770 (9.3%)	78 (7.7%)	0.102	775 (12.5%)	81 (11.2%)	0.302
Congestive heart failure	326 (3.9%)	132 (13.1%)	<.001	312 (5.0%)	102 (14.0%)	<.001
Hyperthyroidism	96 (1.2%)	20 (2.0%)	0.026	18 (0.3%)	≤5 (0.1%)	0.458
Aortic stenosis	118 (1.4%)	41 (4.1%)	<.001	101 (1.6%)	37 (5.1%)	<.001
Hemoglobin	141.0 (129.0–151.0)	128.0 (114.0–143.0)	<.001	141.0 (127.0–152.0)	125.0 (111.0–138.0)	<.001
White blood count	9.4 (7.6–11.8)	11.6 (9.1–15.0)	<.001	9.6 (7.7–12.1)	11.7 (8.9–15.6)	<.001
Sodium	139.0 (137.0–141.0)	139.0 (136.0–141.0)	<.001	139.0 (137.0–141.0)	138.0 (135.0–141.0)	<.001
Potassium	4.0 (3.7–4.4)	4.2 (3.9–4.7)	<.001	4.0 (3.7–4.4)	4.3 (3.9–4.8)	<.001
Glucose	7.7 (6.3–10.5)	9.8 (7.3–14.3)	<.001	7.5 (6.3–9.9)	9.0 (6.8–12.3)	<.001
Urea	6.3 (5.0–8.2)	9.3 (6.6–14.4)	<.001	6.4 (5.0–8.5)	10.2 (7.3–15.2)	<.001
Creatinine	91.0 (77.0–110.0)	120.0 (92.0–171.0)	<.001	92.0 (79.0–113.0)	127.0 (95.0–181.0)	<.001

*Note:* Continuous variables are reported as median (25th percentile–75th percentile); dichotomous variables are reported as *N* (%).

The Kruskal–Wallis test and the Chi-squared test were used to compare continuous and categorical baseline characteristics, respectively, between patients who died within 30 days of admission and those who did not in each of the EFFECT Baseline and EFFECT Follow-up samples.

**Appendix B:** Comparison of baseline covariates between AMI patients in the EFFECT Baseline sample and the EFFECT Follow-up sample

Variable	EFFECT Baseline sample *N* = 9298	EFFECT Follow-up sample *N* = 6932	*p*- value
Death within 30 days of admission	1010 (10.9%)	726 (10.5%)	0.427
Age	69.0 (57.0–78.0)	71.0 (58.0–80.0)	<.001
Female sex	3333 (35.8%)	2563 (37.0%)	0.14
Cardiogenic shock	142 (1.5%)	20 (0.3%)	<.001
Acute congestive heart failure/pulmonary edema	526 (5.7%)	479 (6.9%)	0.001
Systolic blood pressure	146.0 (126.0–168.0)	143.0 (122.0–164.0)	<.001
Diastolic blood pressure	82.0 (70.0—95.0)	80.0 (68.0–92.0)	<.001
Heart rate	80.0 (68.0—98.0)	82.0 (69.0–99.0)	0.005
Respiratory rate	20.0 (18.0—22.0)	20.0 (18.0–22.0)	<.001
Diabetes	2433 (26.2%)	1932 (27.9%)	0.015
Hypertension	4286 (46.1%)	4049 (58.4%)	<.001
Current smoker	3010 (32.4%)	1877 (27.1%)	<.001
Dyslipidemia	2859 (30.7%)	3087 (44.5%)	<.001
Family history of CAD	2828 (30.4%)	2187 (31.5%)	0.122
Cerebrovascular disease/TIA	949 (10.2%)	856 (12.3%)	<.001
Angina	3073 (33.1%)	2099 (30.3%)	<.001
Cancer	285 (3.1%)	116 (1.7%)	<.001
Dementia	368 (4.0%)	391 (5.6%)	<.001
Peptic ulcer disease	515 (5.5%)	347 (5.0%)	0.134
Previous AMI	2143 (23.0%)	1672 (24.1%)	0.111
Asthma	514 (5.5%)	427 (6.2%)	0.088
Depression	676 (7.3%)	695 (10.0%)	<.001
Peripheral vascular disease	712 (7.7%)	595 (8.6%)	0.032
Previous revascularization	848 (9.1%)	856 (12.3%)	<.001
Congestive heart failure	458 (4.9%)	414 (6.0%)	0.003
Hyperthyroidism	116 (1.2%)	19 (0.3%)	<.001
Aortic stenosis	159 (1.7%)	138 (2.0%)	0.187
Hemoglobin	140.0 (127.0–151.0)	139.0 (124.0–151.0)	0.024
White blood count	9.6 (7.7–12.2)	9.8 (7.8–12.4)	0.004
Sodium	139.0 (137.0–141.0)	139.0 (137.0–141.0)	<.001
Potassium	4.1 (3.7–4.4)	4.1 (3.8–4.4)	0.828
Glucose	7.8 (6.4–10.9)	7.6 (6.3–10.3)	<.001
Urea	6.5 (5.0–8.6)	6.6 (5.1–9.1)	<.001
Creatinine	93.0 (78.0–115.0)	94.0 (80.0–119.0)	<.001

*Note:* Continuous variables are reported as median (25th percentile–75th percentile); dichotomous variables are reported as *N* (%).

The Kruskal–Wallis test and the Chi-squared test were used to compare continuous and categorical baseline characteristics, respectively, between patients in the EFFECT Baseline sample and the EFFECT Follow-up sample.

**Appendix C:** Comparison of baseline characteristics between CHF patients who died within 30 days of admission and those who survived for 30 days subsequent to admission in the EFFECT Baseline and Follow-up samples

Variable	EFFECT Baseline sample			EFFECT Follow-up sample		
		
	Death within 30 days: No *N* = 7353	Death within 30 days: Yes *N* = 887	*p*- value	Death within 30 days: No *N* = 6853	Death within 30 days: Yes N = 755	*p*- Value
Age	77.0 (69.0–83.0)	82.0 (74.0–88.0)	<.001	78.0 (70.0–84.0)	83.0 (77.0–88.0)	<.001
Female sex	3692 (50.2%)	465 (52.4%)	0.213	3478 (50.8%)	408 (54.0%)	0.086
Systolic blood pressure	148.0 (128.0–172.0)	130.0 (112.0–152.0)	<.001	146.0 (126.0–169.0)	128.0 (109.0–148.0)	<.001
Heart rate	92.0 (76.0–110.0)	94.0 (78.0–110.0)	0.208	90.0 (73.0–108.0)	93.0 (76.0–111.0)	0.008
Respiratory rate	24.0 (20.0–30.0)	25.0 (20.0–32.0)	<.001	24.0 (20.0–28.0)	24.0 (20.0–30.0)	<.001
Neck vein distension	4062 (55.2%)	455 (51.3%)	0.026	4161 (60.7%)	435 (57.6%)	0.098
S3	728 (9.9%)	57 (6.4%)	<.001	435 (6.3%)	31 (4.1%)	0.015
S4	284 (3.9%)	18 (2.0%)	0.006	192 (2.8%)	9 (1.2%)	0.009
Rales >50% of lung field	752 (10.2%)	151 (17.0%)	<.001	841 (12.3%)	131 (17.4%)	<.001
Pulmonary edema	3766 (51.2%)	452 (51.0%)	0.884	4151 (60.6%)	452 (59.9%)	0.707
Cardiomegaly	2652 (36.1%)	292 (32.9%)	0.065	3043 (44.4%)	329 (43.6%)	0.664
Diabetes	2594 (35.3%)	280 (31.6%)	0.028	2619 (38.2%)	239 (31.7%)	<.001
Cerebrovascular disease/TIA	1161 (15.8%)	213 (24.0%)	<.001	1217 (17.8%)	184 (24.4%)	<.001
Previous AMI	2714 (36.9%)	307 (34.6%)	0.18	2505 (36.6%)	269 (35.6%)	0.617
Atrial fibrillation	2139 (29.1%)	264 (29.8%)	0.677	2417 (35.3%)	297 (39.3%)	0.027
Peripheral vascular disease	950 (12.9%)	132 (14.9%)	0.102	915 (13.4%)	111 (14.7%)	0.303
Chronic obstructive pulmonary disease	1211 (16.5%)	194 (21.9%)	<.001	1518 (22.2%)	229 (30.3%)	<.001
Cirrhosis	52 (0.7%)	11 (1.2%)	0.085	52 (0.8%)	≤5 (0.4%)	0.266
Cancer	814 (11.1%)	136 (15.3%)	<.001	749 (10.9%)	131 (17.4%)	<.001
Left bundle branch block	1082 (14.7%)	150 (16.9%)	0.083	934 (13.6%)	99 (13.1%)	0.694
Hemoglobin	125.0 (111.0–138.0)	120.0 (105.0–136.0)	<.001	123.0 (109.0–137.0)	118.0 (105.0–132.0)	<.001
White blood count	8.9 (7.0–11.4)	10.0 (7.5–12.9)	<.001	8.8 (7.0–11.4)	9.8 (7.6–13.0)	<.001
Sodium	139.0 (136.0–141.0)	138.0 (135.0–141.0)	<.001	139.0 (136.0–142.0)	138.0 (135.0–142.0)	0.001
Potassium	4.2 (3.9–4.6)	4.4 (4.0–4.9)	<.001	4.2 (3.8–4.6)	4.4 (4.0–4.9)	<.001
Glucose	7.5 (6.0–10.7)	7.7 (6.2–10.9)	0.02	7.3 (6.0–10.1)	7.5 (6.1–10.2)	0.158
Urea	8.1 (6.0–11.8)	11.7 (8.1–17.4)	<.001	8.2 (6.0–11.6)	11.4 (7.8–18.3)	<.001

*Note:* Continuous variables are reported as median (25th percentile–75th percentile); dichotomous variables are reported as *N* (%).

The Kruskal–Wallis test and the Chi-squared test were used to compare continuous and categorical baseline characteristics, respectively, between patients who died within 30 days of admission and those who did not in each of the EFFECT Baseline and EFFECT Follow-up samples.

**Appendix D:** Comparison of baseline covariates between CHF patients in the EFFECT Baseline sample and the EFFECT Follow-up sample

Variable	EFFECT Baseline sample (*N* = 8240)	EFFECT Follow-up sample (*N* = 7608)	*p*- value
Death within 30 days of admission	887 (10.8%)	755 (9.9%)	0.083
Age	77.0 (70.0–84.0)	79.0 (70.0–85.0)	<.001
Female sex	4157 (50.4%)	3886 (51.1%)	0.429
Systolic blood pressure	146.0 (126.0–170.0)	144.0 (124.0–167.5)	<.001
Heart rate	92.0 (76.0–110.0)	90.0 (73.0–109.0)	<.001
Respiratory rate	24.0 (20.0–30.0)	24.0 (20.0–28.0)	<.001
Neck vein distension	4517 (54.8%)	4596 (60.4%)	<.001
S3	785 (9.5%)	466 (6.1%)	<.001
S4	302 (3.7%)	201 (2.6%)	<.001
Rales >50% of lung field	903 (11.0%)	972 (12.8%)	<.001
Pulmonary edema	4218 (51.2%)	4603 (60.5%)	<.001
Cardiomegaly	2944 (35.7%)	3372 (44.3%)	<.001
Diabetes	2874 (34.9%)	2858 (37.6%)	<.001
Cerebrovascular disease/TIA	1374 (16.7%)	1401 (18.4%)	0.004
Previous AMI	3021 (36.7%)	2774 (36.5%)	0.793
Atrial fibrillation	2403 (29.2%)	2714 (35.7%)	<.001
Peripheral vascular disease	1082 (13.1%)	1026 (13.5%)	0.511
Chronic obstructive pulmonary disease	1405 (17.1%)	1747 (23.0%)	<.001
Cirrhosis	63 (0.8%)	55 (0.7%)	0.761
Cancer	950 (11.5%)	880 (11.6%)	0.941
Left bundle branch block	1232 (15.0%)	1033 (13.6%)	0.014
Hemoglobin	124.0 (110.0–138.0)	123.0 (109.0–137.0)	0.001
White blood count	9.0 (7.1–11.6)	8.9 (7.0–11.5)	0.062
Sodium	139.0 (136.0–141.0)	139.0 (136.0–142.0)	0.028
Potassium	4.2 (3.9–4.6)	4.2 (3.9–4.6)	0.105
Glucose	7.5 (6.1–10.7)	7.3 (6.0–10.1)	<.001
Urea	8.4 (6.1–12.4)	8.4 (6.2–12.2)	0.635

*Note:* Continuous variables are reported as median (25th percentile–75th percentile); dichotomous variables are reported as *N* (%).

The Kruskal–Wallis test and the Chi-squared test were used to compare continuous and categorical baseline characteristics, respectively, between patients in the EFFECT Baseline sample and the EFFECT Follow-up sample.
